# Dual RNA-Seq of H5N1 Avian Influenza Virus and Host Cell Transcriptomes Reveals Novel Insights Into Host-Pathogen Cross Talk

**DOI:** 10.3389/fmicb.2022.828277

**Published:** 2022-04-12

**Authors:** Qiao Wang, Zixuan Wang, Jin Zhang, Qi Zhang, Maiqing Zheng, Jie Wen, Guiping Zhao, Qinghe Li

**Affiliations:** State Key Laboratory of Animal Nutrition, Institute of Animal Sciences, Chinese Academy of Agricultural Sciences, Beijing, China

**Keywords:** dual RNA sequencing, H5N1, chicken, *CSE1L*, lncRNA

## Abstract

H5N1 avian influenza virus (AIV) is a highly pathogenic influenza virus that poses a substantial threat to poultry production and public health. A comprehensive understanding of host–pathogen interactions for AIV requires knowledge of gene expression changes in both the pathogen and the host upon infection. We report the use of dual RNA sequencing technology to uncover trends in gene expression in H5N1 AIV and chickens (DF1 cells) during the course of infection. The expression of all viral genes increased continuously from 0 to 20 h post infection. We also identified 2,762 differentially expressed host genes during infection. Pathway analysis found that genes related to the signaling pathways of DNA replication, T cell activation, NF-kappa B signaling pathway, and RNA degradation were significantly enriched. We demonstrated that the *cis*-acting lncRNA MSTRG.14019.1 targeted *CSE1L* and may affect virus replication. This study provides a more comprehensive and detailed understanding of host-virus interactions at the RNA level during the course of H5N1 AIV infection.

## Introduction

Influenza A virus (IAV) is a member of the *Orthomyxoviridae* family and is a significant threat to public health. Moreover, highly pathogenic H5N1 avian strains also threaten to cause worldwide pandemics and substantial economic losses (Schrauwen et al., 2014). IAV is a segmented, single-stranded, negative-sense RNA virus, with a genome consisting of eight gene segments that encode at least 11 proteins (Wise et al., 2009). Upon infection by influenza, host cells detect viral RNA through pathogen sensors, and the major gene products of the influenza virus mediate the viral life cycle and modulate cellular processes (Dou et al., 2018).

A comprehensive understanding of host-pathogen interactions requires knowledge of the associated gene expression changes in the pathogen and the host. Dual RNA sequencing (dual RNA-seq) technology allows for the parallel analysis of host and pathogen transcriptomes, which facilitates the detection of gene expression changes in the pathogen and host simultaneously (Westermann et al., 2012). Most importantly, the dual RNA-seq approach allows genes from both host and pathogen to be monitored at different time points throughout infection, from initial contact through to invasion, and finally, the manipulation of the host (Westermann et al., 2016).

Pathogen infection triggers a series of dynamic reactions in the host, and eventually leads to changes in the gene expression patterns of the pathogen and the host. Such changes may lead to the adaptation and tolerance of the pathogen, or may trigger the host immune response to eliminate the pathogen. A comprehensive understanding of host and pathogen transcriptome information will help us to identify virulence factors of new pathogens, pathogen-associated molecular patterns, or new host pathways that target specific pathogens, thus, furthering the understanding of interactions between pathogens and hosts (Baddal et al., 2015; Ranaware et al., 2016).

Avian derived cell lines such as chicken embryonic fibroblasts (DF1) can be used to propagate influenza viruses because they express α-2,3-linked sialic acid receptors, which are preferentially targeted by avian-adapted viruses (Lee et al., 2008). Moreover, immortalized cell lines provide a suitable platform for generating stable cell lines that can be used for virus propagation (Chungu et al., 2021). Here, the dynamic regulation of virus and host gene expression in H5N1 AIV-infected chicken DF1 cells was studied using dual RNA-seq. After H5N1 AIV infection of chicken DF1 cells for 0, 6, 12, and 20 h, the expression of different viral genes was increased. A total of 2,762 differentially expressed host genes were also identified at different time points. We further explained the role of the differentially expressed genes (DEGs) in viral infection using pathway enrichment analyses, and compared those findings to published proteomics or genome-wide RNA interference data to screen key genes that affect viral replication. Notably, the cis-acting lncRNA MSTRG.14019.1 targeted and regulated the influenza promoting gene, *CSE1L*, which may affect virus replication. We also found that the expression of lncRNA MSTRG.14019.1 was significantly increased at 12 h post infection (hpi) with H5N1 AIV, which is contrary to the expression trend of *CSE1L*. Thus, these results suggest that lncRNA MSTRG.14019.1 may inhibit the replication of H5N1 AIV by inhibiting the expression of *CSE1L*.

## Materials and Methods

### Cells and Virus

DF1 cells and Madin–Darby canine kidney (MDCK) cells were cultured in Dulbecco’s modified Eagle’s medium (DMEM) supplemented with 10% fetal bovine serum (FBS, Gibco), 100 μg/ml streptomycin and 100 U/ml penicillin at 37°C, under a humidified atmosphere of 5% CO_2_. The highly pathogenic H5N1 strain A/mallard/Huadong/S/2005 (SY) (Tang et al., 2009) was propagated in 10-day-old specific-pathogen-free embryonic chicken eggs. All experiments involving live viruses were performed in a biosafety cabinet with HEPA filters in a biosafety level 3 laboratory at Yangzhou University, Yangzhou, China.

### Viral Infection and RNA Sample Collection

DF1 cells were incubated in a 6-well plate at 37°C and 5% CO_2_. The cells were cultured until they achieved 90% confluence. Four 6-well plates containing DF1 cells were cultured under the same conditions. The uninfected cells were collected directly and stored in liquid nitrogen. The three remaining plates were infected with 0.1 PFU at 37°C and incubated for 1 h at room temperature. The inoculum was then removed and replaced with DMEM lacking FBS. Cells were collected at 6, 12, and 20 h after infection, and stored in liquid nitrogen. Total RNA was extracted using an RNeasy minikit (Qiagen, Valencia, CA, United States), followed by DNase treatment.

### Strand-Specific Total RNA Library Preparation and Sequencing

All samples had an RNA Integrity Number (RIN) of > 8 (Agilent Technologies, Santa Clara, CA, United States). Starting with 1 μg of DNA-free total RNA extracted from mock- and H5N1-infected DF1 cells, rRNA was first removed through two rounds of selection using a Ribo-Zero rRNA removal kit (Epicenter, Madison WI, United States). The resulting RNA was then used to construct an RNA-seq library using the Illumina Truseq strand-specific library preparation kit (Illumina, Inc., San Diego, CA, United States). To obtain the final sequencing library, 15 cycles of Polymerase Chain Reaction (PCR) were performed using Phusion Hot Start high-fidelity DNA polymerase (Finnzymes, Espoo, Finland). RNA-seq libraries were size selected for the retention of insert fragments between 100 and 300 base pairs (bp). Mature small RNAs (including svRNAs) were not considered in our analysis. For each sample replicate, we obtained ∼30 million paired 50-mer reads (with three biological replicates per sample) using the Illumina Hiseq-2000 platform (Illumina, Inc.).

### Read Mapping and Differential Expression Analysis of RNA-Seq Data

For the chicken host transcriptome analysis, reads were aligned in paired-end mode to a chicken genome (Ensemble gga6.0) using the HISAT2^1^ using the default settings. Reads that were not mapped to Ensemble gga6.0 were mapped to influenza genomes: A/goose/Guangdong/1/1996(H5N1).^2^ Viral reads were mapped to viral genomes using Bowtie2^3^ (Fabozzi et al., 2018). Differential expression was evaluated for each time point using edgeR (Lun et al., 2016). Differential expression was detected using the following thresholds: FDR < 0.05 & |log2FC| ≥ 1.

### Identification of Candidate lncRNAs and Target Gene Prediction

The low-quality reads, adaptor sequences, empty reads, and ribosomal (r)RNA reads were removed from the raw data to obtain high-quality lncRNAs. The read coverage of transcripts was calculated using Stringtie (1.3.3) (Pertea et al., 2015), and those with reads shorter than 200 nt were eliminated. The Coding-Non-Coding-Index (CNCI) (Sun et al., 2013) (score < 0), Coding Potential Calculator (CPC) (Kong et al., 2007) (score < 0), and Pfam-scan (Finn et al., 2016) (*E*-value < 0.001) were used to assess the coding potential of the remaining transcripts. Transcripts identified with coding potential by any of the three tools were filtered out.

The identified lncRNAs were classified by FEELnc (Wucher et al., 2017). According to the location with the corresponding gene, lncRNAs were classified as intergenic or intragenic. The intragenic lncRNAs were further subclassified into four categories: (1) sense intronic lncRNAs, (2) antisense intronic lncRNAs, (3) sense exonic lncRNAs, and (4) antisense exonic lncRNAs.

Differentially expressed lncRNAs (DE-lncRNAs) were selected for target prediction. The cis role of lncRNAs was their action on neighboring target genes (Ørom et al., 2010). The DEGs located within a 100 kb distance of the DE-lncRNAs were selected as potential target genes. The Pearson correlation test was used to calculate the correlation coefficients between lncRNAs and their potential target genes. RNAplex (Tafer and Hofacker, 2008) was used to predict the target genes of tran-acting lncRNAs. RNAplex can quickly identify the possible hybridization sites with the query RNA sequence (lncRNA) in the RNA database (i.e., the target genes of trans-acting lncRNAs).

### Gene Ontology Term Enrichment and Pathway Enrichment Analyses

Gene ontology enrichment and pathway enrichment were conducted using the Database for Annotation, Visualization and Integrated Discovery (DAVID) (Huang et al., 2009).

### Quantitative Real-Time Polymerase Chain Reaction

The efficiency of the knockdown of *CSE1L* expression was confirmed by quantitative real-time PCR. Total RNA was isolated from DF1 cells using TRIzol reagent (Tiangen). One microgram of total RNA per sample was reverse transcribed into cDNA using the FastQuant RT Kit (Tiangen). We next used the ABI Prism 7500 system (Applied Biosystems) with the One Step SYBR PrimeScript RT-PCR Kit II (Takara) to analyze the expression of *CSE1L*. The expression of each gene, relative to that of glyceraldehyde-3-phosphate dehydrogenase, was calculated using the 2^–ΔΔ*ct*^ method.

### RNA Interference

All of the siRNAs used in this study were designed and synthesized by Guangzhou Ruibo (Guangzhou, China). At a confluence of 90% in 6-well plates, DF1 cells were transfected with 100 nM of siRNA targeting the chicken *CSE1L* gene (Gene ID: 395958); siCSE1L, sense 5′-GCAAAGAATCCATCTGTTA-3′). The negative control siRNA was a scrambled siRNA for CSE1L (siNC, sense 5′-GUGAACGAACUCCUUAAUUTT-3′). All siRNAs were transfected into DF1 cells using Lipofectamine 3000 (Life Technologies).

### Infectious Titer of Influenza Virus (TCID50 Assay)

The TCID50 assay was used to evaluate progeny virus production. After transfection with siRNA targeting the *CSE1L* gene or the negative control siRNA, all of the DF1 cells were infected with the SY virus at a multiplicity of infection of 0.1 PFU. At 1 h post infection (1 hpi), the medium was replaced with DMEM lacking fetal bovine serum. Conditioned medium was then collected at 12 and 24 hpi to measure viral titers. Viral titers were determined using the agglutination assay after growth in MDCK cells. The MDCK cells were seeded in 96-well plates and infected after reaching 85% confluence. Cells were washed twice with phosphate-buffered saline (PBS) and infected with a series of virus dilutions, and incubated for 72 h, as described above. Agglutination assays were performed in round-bottomed 96-well plates using 1% chicken red blood cells in PBS.

### Statistical Analysis

The viral titers were calculated as the means ± standard deviations from three independent experiments. Independent-samples *t*-tests were used to analyze the TCID50. For all tests, a *p* ≤ 0.05 was considered statistically significant.

## Results

### Overview of the Dual RNA-Seq Data From H5N1-Infected Chicken DF1 Cells

**FIGURE 1:**
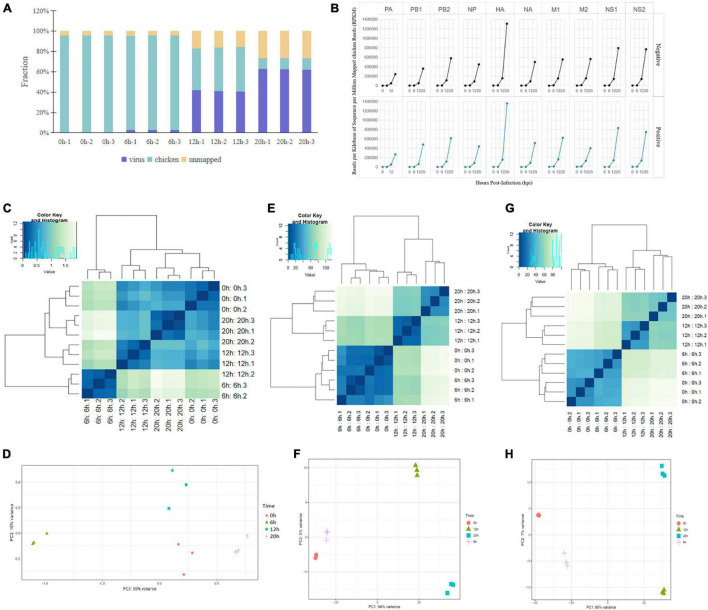
Dual RNA-seq data of H5N1-infected chicken DF1 cells. **(A)** Total RNA-seq read counts were mapped by read fraction distribution. **(B)** Temporal expression of H5N1 influenza viral RNAs. Negative- and positive-stranded RNAs of each viral segment expressed as reads per kilobase of sequence per million mapped reads normalized to total host reads. **(C,E,G)** PCA of the differentially expressed influenza virus RNAs, chicken mRNAs, and chicken lncRNAs at four time points post infection. **(D,F,H)** Cluster analysis of the differentially expressed influenza virus RNAs, chicken mRNAs, and chicken lncRNAs at four time points post infection.

We infected chicken DF1 cells with the highly pathogenic H5N1 strain, A/mallard/Huadong/S/2005 (SY). Total RNA was extracted from virus-infected cells at 0, 6, 12, and 20 hpi, and subjected to a dual strand-specific RNA-seq strategy using the Illumina HiSeq instrument. Figure 1A shows the distribution of host and viral read counts for each sample. After viral infection, the viral read counts increased gradually from 0.02 to 62.44%. Conversely, the number of read counts decreased gradually from 95.71 to 10.81%. Following viral infection, the unmatched read counts increased continuously (Figure 1A). The expression of negative-strand RNAs (vRNAs) versus positive-strand RNAs (cRNA/mRNA) from each gene segment was evaluated by normalizing the read coverage of viral gene segments to the total number of chicken reads (Figure 1B). To understand the different characteristics between different time points after infection, we conducted cluster analyses and principal component analyses (PCA) of the differentially expressed influenza virus RNA, and chicken mRNA and lncRNA. Hierarchical clustering revealed that three virus infection groups and the control group were well distinguished in virus RNA and chicken mRNA and lncRNA, with all subjects correctly classified (Figures 1D,F,H). PCA also revealed distinct expression signatures of all three RNA types at different time points after infection (Figures 1C,E,G).

### RNA-Seq Identified Differentially Expressed mRNAs

**FIGURE 2:**
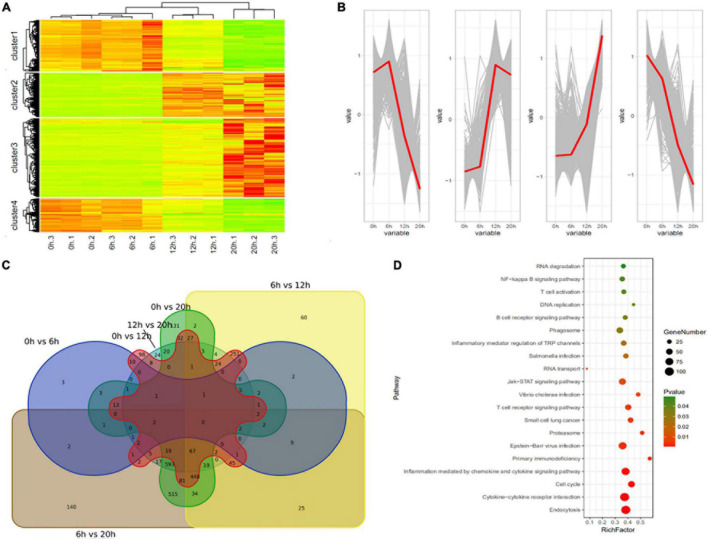
Differentially expressed genes in H5N1-infected chicken DF1 cells. **(A)** Clustering analysis of differential gene expression patterns at 0, 6, 12, and 20 hpi. **(B)** The trends in differential gene expression patterns at 0, 6, 12, and 20 hpi. **(C)** Venn diagrams of overlapping DEGs between different time points. **(D)** Pathway enrichment analysis of the DEGs.

A total of 2,762 genes were differentially regulated following H5N1 AIV infection in chicken DF1 cells. We then analyzed the number of DEGs, including up-regulated genes and down-regulated genes, between each pair of time points following infection (Table 1). Heat maps and line charts were generated to aid in the visualization of gene expression patterns (Figures 2A,B). The results also showed that the DEGs mainly conformed to four expression patterns: (1) they were first decreased and then increased, (2) first increased and then decreased, (3) continuously increased, and (4) continuously decreased (Figures 2A,B). Comparative analyses between six groups of DEGs (0 hpi vs. 6 hpi, 0 hpi vs. 12 hpi, 0 hpi vs. 20 hpi, 6 hpi vs. 12 hpi, 6 hpi vs. 20 hpi and 12 hpi vs. 20 hpi) were performed (Figure 2C). There were several DEGs shared between different groups, however, many more genes were expressed at different time points following infection (Figure 2C and Supplementary Table 1). Next, we performed functional classification analyses of the DEGs using DAVID Bioinformatics Resources. Gene Ontology (GO) enrichment of the DEGs included positive regulation of intrinsic apoptotic signaling pathway, DNA replication, nuclear envelope, and ATP-dependent helicase activity (Table 2). The DEGs related to pathways for DNA replication, T cell activation, NF-kappa B signaling, and RNA degradation were also significantly enriched (Figure 2D). We also analyzed the pathway enrichment of DEGs at different infection time points (Supplementary Table 2).

**TABLE 1:** Differentially expressed genes at different time points post infection in chicken DF1 cells.

Group	All	Up	Down
0 h vs. 6 h	67	29	38
0 h vs. 12 h	1,144	835	309
0h vs. 20 h	2,028	900	1,128
6h vs. 12 h	1,039	774	265
6 h vs. 20 h	2,037	881	1,156
12 h vs. 20 h	821	274	547

**TABLE 2:** Gene ontology enrichment of differentially expressed genes.

GO term	Gene number	*P*-value
**Biological process**		
Positive regulation of intrinsic apoptotic signaling pathway	7	0.0033
Regulation of DNA-dependent DNA replication initiation	4	0.01
DNA replication	15	0.011
Negative regulation of insulin receptor signaling pathway	8	0.016
Maturation of SSU-rRNA from tricistronic rRNA transcript (SSU-rRNA, 5.8S rRNA, LSU-rRNA)	8	0.016
Hormone metabolic process	4	0.022
Cellular response to insulin stimulus	9	0.023
Protein glycosylation	14	0.025
ER-associated ubiquitin-dependent protein catabolic process	11	0.028
Nuclear migration	4	0.04
Cellular response to glucose stimulus	5	0.041
Protein folding	18	0.042
Protein homotetramerization	9	0.043
Cell division	17	0.045
Protein export from nucleus	6	0.049
**Molecular function**		
Mitochondrion	122	0.000039
Extracellular exosomes	254	0.000075
Myelin sheath	31	0.00043
Centrosome	52	0.0011
Cytosol	129	0.0025
Membrane	114	0.0042
Acrosomal vesicle	9	0.0058
Sperm flagellum	6	0.0076
Ubiquitin ligase complex	15	0.0078
Pwp2p-containing subcomplex of 90S preribosome	4	0.01
Endoplasmic reticulum lumen	11	0.013
Midbody	16	0.021
Nuclear envelope	15	0.033
Focal adhesion	47	0.037
Golgi apparatus	62	0.04
Intracellular ribonucleoprotein complex	10	0.042
Kinetochore	11	0.048
**Cellular component**		
Poly(A) RNA binding	109	0.003
Collagen binding	6	0.004
ATP binding	153	0.018
Metalloendopeptidase activity	18	0.018
ATP-dependent helicase activity	5	0.026
GTP binding	45	0.03
GDP binding	10	0.032
Metallopeptidase activity	6	0.035

### RNA-Seq Identified Differentially Expressed lncRNAs

**FIGURE 3:**
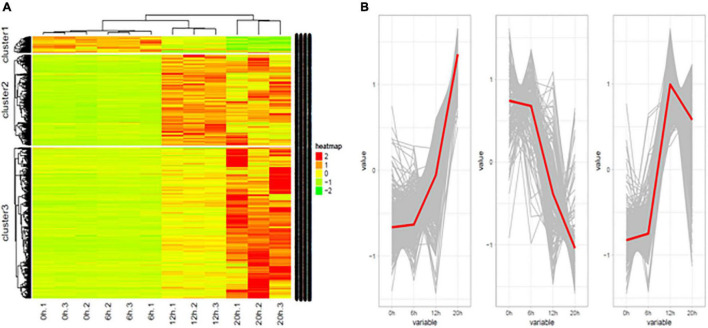
Differentially expressed lncRNAs in H5N1-infected chicken DF1 cells. **(A)** Cluster analysis of the expression patterns of differential lncRNAs at 0, 6, 12, and 20 hpi. **(B)** The trends in differential lncRNAs expression patterns at 0, 6, 12, and 20 hpi.

A total of 1,416 lncRNAs were found to be differentially expressed between the uninfected group and the infected group, or between groups at different time points post infection (Table 3). Similar to the mRNA results, heat maps were generated to aid the visualization of the lncRNA expression patterns (Figure 3). The results showed that the DE-lncRNAs exhibited three main expression patterns: (1) they were first increased and then decreased, (2) continuously increased, and (3) continuously decreased (Figure 3).

**TABLE 3:** Differentially expressed lncRNAs.

Group	All	Up	Down
0 h vs. 6 h	29	17	12
0 h vs. 12 h	599	483	116
0 h vs. 20 h	960	459	501
6 h vs. 12 h	520	402	118
6 h vs. 20 h	922	436	486
12 h vs. 20 h	320	148	172

### Target Genes of lncRNAs

**FIGURE 4:**
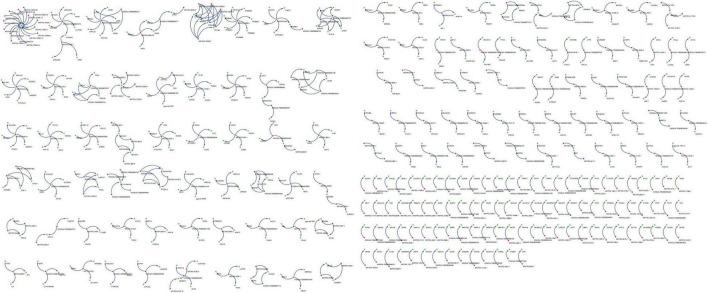
Targeted regulatory relationships of lncRNA-mRNA. Green dots represent differentially expressed genes and blue dots represent differentially expressed lncRNAs.

A total of 6,586 and 4,788 pairs of regulatory relationships were identified by the cis- and trans- predictive analyses, respectively (Supplementary Tables 3, 4). There were 1,128 pairs of targeted regulatory relationships between the DE-lncRNAs and their cis- and trans-target DEGs (Supplementary Table 5). The interaction networks of the DE-lncRNAs and their cis- and trans-target DEGs were also constructed (Supplementary Table 5 and Figure 4). The mapping of lncRNA-mRNA relationships showed that lncRNAs target mRNAs in a variety of ways. For example, 16 lncRNAs, including lncRNA MSTRG.10545.15 and lncRNA MSTRG.10545.20 simultaneously target SNAP47 mRNA. Additionally, lncRNA ENGALT00000081479 binds to six genes, including P4HB and ARHGDIA, and many other lncRNAs target specific genes (Figure 4). GO enrichment of the cis- and trans-target genes of DE-lncRNAs included peptide antigen binding, regulation of T cell mediated immunity, the MHC class I protein complex, and positive regulation of T cell-mediated immunity (Supplementary Table 6). All mRNAs targeted by the differentially expressed lncRNAs were analyzed for pathway enrichment. The results revealed that 25 pathways were significantly enriched, while 16 pathways, including antigen processing and presentation, natural killer cell-mediated cytotoxicity, phagosome, and the Fc epsilon RI signaling pathway were implicated in the immune system or diseases (Table 4).

**TABLE 4:** Pathway enrichment of the target genes of differentially expressed lncRNAs.

Term	Count	*P*-value
Allograft rejection	43	1.37E-15
Graft-versus-host disease	40	2.17E-14
Type I diabetes mellitus	42	3.66E-14
Viral myocarditis	59	2.36E-13
Autoimmune thyroid disease	41	1.41E-12
Antigen processing and presentation	43	6.97E-09
Natural killer cell mediated cytotoxicity	39	1.12E-07
Cell adhesion molecules (CAMs)	52	6.65E-07
Phagosome	60	7.82E-07
Herpes simplex infection	54	4.49E-06
Epstein-Barr virus infection	57	1.18E-05
Phenylalanine metabolism	12	2.94E-05
HTLV-I infection	69	8.69E-05
Asthma	12	0.000165336
Phenylalanine, tyrosine and tryptophan biosynthesis	6	0.000592853
Fat digestion and absorption	14	0.000640793
Ubiquinone and other terpenoid-quinone biosynthesis	8	0.001249166
MicroRNAs in cancer	39	0.001327914
Staphylococcus aureus infection	15	0.001642345
Phosphonate and phosphinate metabolism	7	0.001645979
Cardiac muscle contraction	24	0.002032595
Leishmaniasis	18	0.002745978
Tropane, piperidine and pyridine alkaloid biosynthesis	5	0.003181575
Amyotrophic lateral sclerosis (ALS)	14	0.003277039
Fc epsilon RI signaling pathway	17	0.0042699

### LncRNA MSTRG.14019.1 Targeting *CSE1L* Inhibits the Replication of H5N1 AIV

**FIGURE 5:**
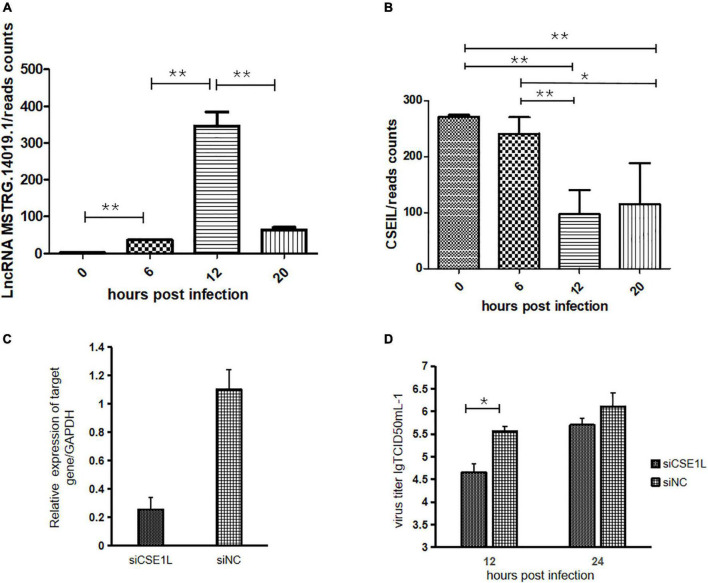
LncRNA MSTRG.14019.1 targeting to *CSE1L* inhibits the replication of H5N1 AIV. **(A)** Expression levels of lncRNA MSTRG.14019.1 at different time points after H5N1 AIV infection. **(B)** Expression levels of *CSE1L* at different time points after H5N1 AIV infection. **(C)** The expression of *CSE1L* was dramatically reduced in specific siRNA-treated DF1 cells. **(D)** Progeny virus titers after transfection of DF1 cells with siCSE1L and infection with H5N1 AIV for 12 or 24 h. *p < 0.05 and **p < 0.01.

The analysis of DE-lncRNAs and their target genes revealed that lncRNA MSTRG.14019.1 targeted *CSE1L*. LncRNA MSTRG.14019.1 is located on the positive chain of chromosome 20 and is located upstream of *CSE1L*. The distance between *CSE1L* and lncRNA MSTRG.14019.1 was 75,117 bp, thus, indicating cis regulation. Compared to the uninfected group, the expression level of lncRNA MSTRG.14019.1 was significantly increased at 12 hpi, but it was significantly decreased at 20 hpi (Figure 5A). Contrary to the expression trend of lncRNA MSTRG.14019.1, the expression of *CSE1L* decreased significantly at 12 hpi (Figure 5B).

To study the role of *CSE1L* in the virus life cycle, we analyzed the effect of *CSE1L* downregulation on H5N1 AIV infection using small interfering RNA (siRNA)-mediated silencing. Real-time PCR confirmed that *CSE1L* expression was dramatically reduced in siRNA-treated DF1 cells, but not in cells treated with the scrambled control siRNA (Figure 5C). Subsequently, chicken DF1 cells treated with siRNA targeting *CSE1L* or with scrambled siRNA were then infected with H5N1 AIV. Culture supernatants were collected at 12 and 24 hpi, and titrated onto MDCK cells. As shown in Figure 5D, knockdown of *CSE1L* decreased the virus titer relative to that observed in scrambled siRNA-treated DF1 cells.

## Discussion

For many years, studies of cellular host responses to viral infections have focused on global changes at the mRNA level. More recently, dual RNA-seq has been used to simultaneously analyze the changes in host and pathogen mRNA expression profiles throughout the course of infection (Baddal et al., 2015; Fabozzi et al., 2018; Mika-Gospodorz et al., 2020; Pisu et al., 2020; Zhang et al., 2020; Karamitros et al., 2021; Penaranda et al., 2021). For example, Pisu et al. (2020) explored the *in vivo* molecular dynamics of Mtb infection by performing dual RNA-seq on Mycobacterium tuberculosis-infected, ontogenetically distinct macrophage lineages isolated directly from murine lungs. Similarly, the transcriptional response of intracellular *Pseudomonas aeruginosa* associated with urinary tract infections and their corresponding host cells were defined using simultaneous dual RNA-seq transcriptional profiling (Penaranda et al., 2021). Here, we used dual RNA-seq to characterize novel mechanisms of the interactions between the chicken DF1 cell line and H5N1 AIV, including those at the mRNA and lncRNA levels.

Through the transcriptome study of the interaction between H5N1 AIV and chicken DF1 cells, the dynamic change trend of virus and host genes at different infection time points has been more comprehensively detected, and lncRNA and mRNA with a targeted regulation relationship have also been found, which is helpful to analyze the virus host interaction and the new mechanism of host affecting avian influenza virus (AIV). In this study, H5N1 AIV infection resulted in 2762 DEGs in chicken DF1 cells. Pathway enrichment analyses of the DEGs identified significantly enriched pathways related to DNA replication, T cell activation, NF-kappa B signaling, and RNA degradation (Figure 2D). We further analyzed the pathway enrichment of DEGs at different time points post infection. The results showed that the inflammation mediated by chemokines and cytokine signaling pathways was significantly enriched in the DEGs at all infection time points, especially in the early stages of infection. The results revealed that during viral infection, cytokines and chemokines respond quickly to viral stimuli, thus, indicating that cytokines and chemokines, and their related signaling pathways play an important role in the process of H5N1 AIV infection, and may be the main antiviral factors during the early stages of viral infection. It has been reported that H5N1 AIV can significantly increase the levels of cytokines and chemokines in host cells compared with that of H1N1 and H7N2 influenza viruses (Huo et al., 2018). Changes in cytokine and chemokine responses during AIV infection are crucial to the pathogenesis of avian influenza in chickens (Kuribayashi et al., 2013; Luo et al., 2018). With increases in infection time, some classical signaling pathways, such as the B cell receptor signaling pathway, T cell receptor signaling pathway, NF-κB signaling pathway, and other important immune-related signaling pathways, became gradually active. In addition, we found that proteasome signaling pathway was also significantly enriched in multiple groups of DEGs (0 hpi vs. 20 hpi, 12 hpi vs. 20 hpi, and 6 hpi vs. 12 hpi). Viruses confiscate cellular components of the proteasome to facilitate many aspects of the infectious cycle (van Tol et al., 2017; Schneider et al., 2021).

The DEGs and pathways identified in this study were compared to other hosts infected with influenza virus. Smith et al. compared gene expression and signaling pathways in chicken ileum and lung samples infected with AIV (Smith et al., 2015). They found that the most significantly enriched pathways in ileum were the metabolic-related pathways, such as phosphoridylinositol signaling, axon guidance, and the PPAR signaling pathway, while in lung, the most significantly enriched pathways included antigen processing and presentation, T cell receptor signaling, and primary immunodeficiency, thus, indicating that the response of different tissues to viral infection was quite different. Therefore, it is important to select appropriate tissues or cells in studies of immunity following viral infection. Chicken DF1 cells were used in this study and the signaling pathways of DEGs affected by viral infection were the same as those found by Smith (Smith et al., 2015) in chicken lung tissue infected by AIV, thus, indicating that DF1 cells are a suitable chicken cell line to study AIV infection. A total of 2,279 genes were differentially expressed in lung tissues of crows infected or not with H5N1 AIV (A/Crow/India/11TI11/2011) (Vijayakumar et al., 2015). Consistent with our results, cytokine-cytokine receptor interaction, Influenza A, chemokine signaling, TNF signaling, NF-kappa B signaling, and Jak-STAT signaling pathways were significantly enriched, suggesting that chickens and wild birds, such as crows, have many similarities in the mechanisms by which they respond to AIV infection.

Viruses rely on host cellular functions to replicate, and thus, they hijack and rewire the host cell machinery for their own needs. We carried out research on the interactions between H5N1 AIV proteins and host proteins to improve our understanding of the viral life cycle, and to assist in identifying host resistance mechanisms. A total of 621 host proteins were obtained by affinity purification mass spectrometry (Li et al., 2016; Wang et al., 2016, 2021). By comparing the 621 host interaction proteins with the 2762 DEGs identified from the transcriptome, 129 DEGs were found among the host proteins to interact with viral proteins (Supplementary Table 7). The host proteins interacting with virus proteins and the DEGs may be the key factors affecting viral replication; thus, the overlapping genes should be focused more intently. These overlapping genes not only interacted with viral proteins, but also exhibited significant changes at the gene expression level after viral infection. Thus, those genes may be important for viral replication.

Several genes have been reported to be involved in viral infection. The chaperonin containing TCP1 complex (CCT) is a multi-subunit complex that can regulate protein folding (Gestaut et al., 2019). T-complex protein 1 subunit epsilon (CCT5) interacts with RNA polymerase NS5B of the hepatitis C virus (Fislová et al., 2010). CCT5 was also found to interact with PA of H5N1 AIV, and is at the core of the virus host interaction network (Wang et al., 2016). The Ras-GTPase-activating protein SH3-domain-binding protein 1 (G3BP1) could also play an important role in the host anti-viral response through associating with RIG-I signaling. G3BP1 formed a complex with RNF125 and RIG-I, leading to decreased RNF125 expression via its auto-ubiquitination, which could inhibit the replication of Sendai virus and vesicular stomatitis virus (Yang et al., 2019). G3BP1 also synergized with RIG-I to induce the expression of the important anti-viral cytokine, IFN-β (Kim et al., 2019).

LncRNAs have been identified to regulate innate antiviral responses via different targets and in various ways, including regulating the activity of RNA binding proteins, and the translation and degradation of host mRNA (Atianand et al., 2017; Wang et al., 2017). In this study, we identified the differently expressed lncRNAs in chicken DF1 cells during H5N1 AIV infection. A total of 1,416 lncRNAs were differentially expressed, and 6,586 and 4,788 pairs of cis- and trans- acting relationships were obtained, respectively (Supplementary Tables 3, 4).

GO and pathway enrichment of the target genes of differentially expressed lncRNAs were analyzed. In terms of the biological processes identified in the GO analyses, genes associated with peptide antigen binding, regulation of T cell mediated immunity, MHC class I protein complexes, and the positive regulation of T cell-mediated immunity were significantly enriched (Supplementary Table 6). Pathway enrichment analysis revealed that 16 of the 25 significantly enriched signaling pathways were related to immunity or disease, such as antigen processing and presentation, natural killer cell mediated cytotoxicity, phagosomes, and Fc epsilon RI signaling pathways (Table 4). These results showed that lncRNAs played an important role in regulating AIV infection and/or host immunity. Collectively, these results provided a subset of lncRNAs that might play important roles in the pathogenesis of influenza viruses and add the lncRNAs to the vast repertoire of host factors used by IAV for infection and persistence.

Chrome aggregation 1 like (*CSE1L*) was first identified in screening tests for drug resistance in breast cancer cells. Mutations in the *CSE1L* homolog in yeast lead to chromosomal segregation abnormalities and mitotic block (Xiao et al., 1993). The N-terminal domain of *CSE1L* is homologous to the RAN binding domain of Importin β, and *CSE1L* is responsible for the nucleation of Importin α to realize the reuse of Importin α. The exudation and reuse of Importin α affects the exudation and transport of a variety of proteins, including host transcription factors, endogenous proteins, and invading viral proteins (Döhner et al., 2018). In the presence of RanGTP, *CSE1L* formed complexes with Importin-α and RanGTP and nucleated. In cytoplasm, the *CSE1L*/Importin-α/RangTP complex is dissociated with the assistance of such proteins as RanBP1 and RanGAP1. Importin-α is dissociated from the complex and participates in the next round of nucleation. *CSE1L* can transport macromolecules out of the nucleus through importin dependent and independent pathways. For example, *CSE1L* can be directly responsible for the transport of homologous recombinant protein RAD51 from the nucleus to the cytoplasm (Okimoto et al., 2015). In this study, we found that cis-acting lncRNA MSTRG.14019.1 targeted *CSE1L*, and the *CSE1L* gene interacted with the NP protein of H5N1 AIV (Wang et al., 2021). More importantly, the expression of lncRNA MSTRG.14019.1 increased significantly at 12 hpi, while the expression of *CSE1L* decreased significantly at the same time point. By transfecting a *CSE1L*-specific siRNA into DF1 cells, we found that the viral replication efficiency decreased significantly after the expression of *CSE1L* was reduced, indicating that *CSE1L* can promote the replication of H5N1 AIV. Based on the above results, we hypothesize that cis-acting lncRNA MSTRG.14019.1 down regulates the expression of *CSE1L*, and inhibits the replication of H5N1 AIV.

The present study is the first to characterize both the host and virus transcriptomes of H5N1-infected chicken DF1 cells at different infection time points. We revealed the trends in expression of virus and host genes, and analyzed the pathway enrichment of host DEGs. We also mapped the regulatory networks of differential lncRNAs and their target genes during viral infection, and found that the cis-acting lncRNA MSTRG.14019.1 targeted *CSE1L* and may affect virus replication. In conclusion, our data highlight the importance of dual RNA-seq in studying host pathogen interactions and identifying novel elements that may contribute to influenza biology and to RNA-based immune responses.

## Data Availability Statement

The data presented in the study are deposited in the Genome Sequence Archive repository (https://ngdc.cncb.ac.cn/gsa/), accession number CRA006311.

## Author Contributions

QW, QL, and GZ conceived, designed the experiments, and wrote the manuscript. QW, ZW, JZ, and QZ performed the experiments. QW and QL analyzed the data. QW, ZW, JZ, MZ, JW, and GZ contributed to reagents, materials, and analysis tools. All authors contributed to the article and approved the submitted version.

## Conflict of Interest

The authors declare that the research was conducted in the absence of any commercial or financial relationships that could be construed as a potential conflict of interest.

## Publisher’s Note

All claims expressed in this article are solely those of the authors and do not necessarily represent those of their affiliated organizations, or those of the publisher, the editors and the reviewers. Any product that may be evaluated in this article, or claim that may be made by its manufacturer, is not guaranteed or endorsed by the publisher.

## Footnotes

1
https://ccb.jhu.edu/software/hisat2/index.shtml

2
https://www.ncbi.nlm.nih.gov/genome/genomes/10290?

3
http://bowtiebio.sourceforge.net/bowtie2/index.shtml

## Supplementary Material

Supplementary Table 1 Comparative analyses between the six groups of DEGs.

Supplementary Table 2 Pathway enrichment of DEGs at different infection time points.

Supplementary Table 3 Cis-target genes of differentially expressed LncRNAs.

Supplementary Table 4 Trans-target genes of differentially expressed LncRNAs.

Supplementary Table 5 Details of 1,128 pairs of targeted regulatory relationships between the DE-lncRNAs and their cis- and trans-target DEGs.

Supplementary Table 6 Gene ontology enrichment of the target DEGs of DE-LncRNAs.

Supplementary Table 7 Common genes from dual RNA-seq and AP-MS.

## References

[B1] Atianand M. K. Caffrey D. R. Fitzgerald K. A. (2017). Immunobiology of long noncoding RNAs. *Annu Rev. Immunol.* 35 177–198. 10.1146/annurev-immunol-041015-055459 28125358 PMC6449690

[B2] Baddal B. Muzzi A. Censini S. Calogero R. A. Torricelli G. Guidotti S. (2015). Dual RNA-seq of nontypeable *Haemophilus influenzae* and host cell transcriptomes reveals novel insights into host-pathogen cross talk. *mBio* 6:e01765. 10.1128/mBio.01765-15 26578681 PMC4659474

[B3] Chungu K. Park Y. H. Woo S. J. Lee B. Rengaraj D. Lee H. J. (2021). Establishment of a genetically engineered chicken DF-1 cell line for efficient amplification of influenza viruses in the absence of trypsin. *BMC Biotechnol.* 21:2. 10.1186/s12896-020-00663-6 33413322 PMC7792337

[B4] Döhner K. Ramos-Nascimento A. Bialy D. Anderson F. Hickford-Martinez A. Rother F. (2018). Importin α1 is required for nuclear import of herpes simplex virus proteins and capsid assembly in fibroblasts and neurons. *PLoS Pathog.* 14:e1006823. 10.1371/journal.ppat.1006823 29304174 PMC5773220

[B5] Dou D. Revol R. Östbye H. Wang H. Daniels R. (2018). Influenza a virus cell entry replication, virion assembly and movement. *Front. Immunol.* 9:1581. 30079062 10.3389/fimmu.2018.01581 PMC6062596

[B6] Fabozzi G. Oler A. J. Liu P. Chen Y. Mindaye S. Dolan M. A. (2018). Strand-specific dual RNA sequencing of bronchial epithelial cells infected with influenza a/h3n2 viruses reveals splicing of gene segment 6 and novel host-virus interactions. *J. Virol.* 92:e00518. 10.1128/JVI.00518-18 29976658 PMC6096831

[B7] Finn R. D. Coggill P. Eberhardt R. Y. Eddy S. R. Mistry J. Mitchell A. L. (2016). The Pfam protein families database: towards a more sustainable future. *Nucleic Acids Res.* 44 D279–D285. 10.1093/nar/gkv1344 26673716 PMC4702930

[B8] Fislová T. Thomas B. Graef K. M. Fodor E. (2010). Association of the influenza virus RNA polymerase subunit PB2 with the host chaperonin CCT. *J. Virol.* 84 8691–8699. 10.1128/JVI.00813-10 20573828 PMC2919027

[B9] Gestaut D. Roh S. H. Ma B. Pintilie G. Joachimiak L. A. Leitner A. (2019). The chaperonin TRiC/CCT associates with prefoldin through a conserved electrostatic interface essential for cellular proteostasis. *Cell* 177 751–765. 10.1016/j.cell.2019.03.012 30955883 PMC6629582

[B10] Huang D. W. Sherman B. T. Lempicki R. A. (2009). Systematic and integrative analysis of large gene lists using DAVID bioinformatics resources. *Nat. Protoc.* 4 44–57. 10.1038/nprot.2008.211 19131956

[B11] Huo C. Xiao K. Zhang S. Tang Y. Wang M. Qi P. (2018). H5N1 influenza a virus replicates productively in pancreatic cells and induces apoptosis and pro-inflammatory cytokine response. *Front. Cell Infect. Microbiol.* 8:386. 10.3389/fcimb.2018.00386 30460207 PMC6232254

[B12] Karamitros T. Pogka V. Papadopoulou G. Tsitsilonis O. Evangelidou M. Sympardi S. (2021). Dual RNA-seq enables full-genome assembly of measles virus and characterization of host-pathogen interactions. *Microorganisms* 9:1538. 10.3390/microorganisms9071538 34361973 PMC8303570

[B13] Kim S. S.-Y. Sze L. Liu C. Lam K.-P. (2019). The stress granule protein G3BP1 binds viral dsRNA and RIG-I to enhance interferon-β response. *J. Biological. Chem.* 294 6430–6438. 10.1074/jbc.RA118.005868 30804210 PMC6484135

[B14] Kong L. Zhang Y. Ye Z. Q. Liu X. Q. Zhao S. Q. Wei L. (2007). CPC: assess the protein-coding potential of transcripts using sequence features and support vector machine. *Nucleic Acids Res.* 35 W345–W349. 10.1093/nar/gkm391 17631615 PMC1933232

[B15] Kuribayashi S. Sakoda Y. Kawasaki T. Tanaka T. Yamamoto N. Okamatsu M. (2013). Excessive cytokine response to rapid proliferation of highly pathogenic avian influenza viruses leads to fatal systemic capillary leakage in chickens. *PLoS One* 8:e68375. 10.1371/journal.pone.0068375 23874602 PMC3706397

[B16] Lee C. W. Jung K. Jadhao S. J. Suarez D. L. (2008). Evaluation of chicken-origin (DF-1) and quail-origin (QT-6) fibroblast cell lines for replication of avian influenza viruses. *J. Virol. Methods* 153 22–28. 10.1016/j.jviromet.2008.06.019 18638503

[B17] Li Q. Yuan X. Wang Q. Chang G. Wang F. Liu R. (2016). Interactomic landscape of PA-X-chicken protein complexes of H5N1 influenza a virus. *J. Proteom.* 148 20–25. 10.1016/j.jprot.2016.07.009 27422376

[B18] Lun A. T. L. Chen Y. Smyth G. K. (2016). “it’s de-licious: a recipe for differential expression analyses of RNA-seq experiments using quasi-likelihood methods in edger,” in *Statistical Genomics: Methods and Protocols*, eds Mathé E. Davis S. (New York, NY: Springer), 391–416. 10.1007/978-1-4939-3578-9_19 27008025

[B19] Luo C. Liu J. Qi W. Ren X. Lu R. Liao M. (2018). Dynamic analysis of expression of chemokine and cytokine gene responses to H5N1 and H9N2 avian influenza viruses in DF-1 cells. *Microbiol. Immunol.* 62 327–340. 10.1111/1348-0421.12588 29577370

[B20] Mika-Gospodorz B. Giengkam S. Westermann A. J. Wongsantichon J. Kion-Crosby W. Chuenklin S. (2020). Dual RNA-seq of *Orientia tsutsugamushi* informs on host-pathogen interactions for this neglected intracellular human pathogen. *Nat. Commun.* 11:3363. 10.1038/s41467-020-17094-8 32620750 PMC7335160

[B21] Okimoto S. Sun J. Fukuto A. Horikoshi Y. Matsuda S. Matsuda T. (2015). hCAS/CSE1L regulates RAD51 distribution and focus formation for homologous recombinational repair. *Genes Cells* 20 681–694. 10.1111/gtc.12262 26123175

[B22] Ørom U. A. Derrien T. Beringer M. Gumireddy K. Gardini A. Bussotti G. (2010). Long noncoding RNAs with enhancer-like function in human cells. *Cell* 143 46–58. 20887892 10.1016/j.cell.2010.09.001 PMC4108080

[B23] Penaranda C. Chumbler N. M. Hung D. T. (2021). Dual transcriptional analysis reveals adaptation of host and pathogen to intracellular survival of *Pseudomonas aeruginosa* associated with urinary tract infection. *PLoS Pathog* 17:e1009534. 10.1371/journal.ppat.1009534 33901267 PMC8102004

[B24] Pertea M. Pertea G. M. Antonescu C. M. Chang T. C. Mendell J. T. Salzberg S. L. (2015). StringTie enables improved reconstruction of a transcriptome from RNA-seq reads. *Nat. Biotechnol.* 33 290–295. 10.1038/nbt.3122 25690850 PMC4643835

[B25] Pisu D. Huang L. Grenier J. K. Russell D. G. (2020). Dual RNA-seq of mtb-infected macrophages in vivo reveals ontologically distinct host-pathogen interactions. *Cell Rep.* 30 335–350. 10.1016/j.celrep.2019.12.033 31940480 PMC7032562

[B26] Ranaware P. B. Mishra A. Vijayakumar P. Gandhale P. N. Kumar H. Kulkarni D. D. (2016). Genome wide host gene expression analysis in chicken lungs infected with avian influenza viruses. *PLoS One* 11:e0153671. 10.1371/journal.pone.0153671 27071061 PMC4829244

[B27] Schneider S. M. Lee B. H. Nicola A. V. (2021). Viral entry and the ubiquitin-proteasome system. *Cell. Microbiol.* 23:e13276. 10.1111/cmi.13276 33037857

[B28] Schrauwen E. J. A. de Graaf M. Herfst S. Rimmelzwaan G. F. Osterhaus A. D. Fouchier R. A. (2014). Determinants of virulence of influenza a virus. *Eur. J. Clin. Microbiol. Infect. Dis.* 33 479–490. 10.1007/s10096-013-1984-8 24078062 PMC3969785

[B29] Smith J. Smith N. Yu L. Paton I. R. Gutowska M. W. Forrest H. L. (2015). A comparative analysis of host responses to avian influenza infection in ducks and chickens highlights a role for the interferon-induced transmembrane proteins in viral resistance. *BMC Genomics* 16:574. 10.1186/s12864-015-1778-8 26238195 PMC4523026

[B30] Sun L. Luo H. Bu D. Zhao G. Yu K. Zhang C. (2013). Utilizing sequence intrinsic composition to classify protein-coding and long non-coding transcripts. *Nucleic Acids Res.* 41:e166. 10.1093/nar/gkt646 23892401 PMC3783192

[B31] Tafer H. Hofacker I. L. (2008). RNAplex: a fast tool for RNA-RNA interaction search. *Bioinformatics* 24 2657–2663. 10.1093/bioinformatics/btn193 18434344

[B32] Tang Y. Wu P. Peng D. Wang X. Wan H. Zhang P. (2009). Characterization of duck H5N1 influenza viruses with differing pathogenicity in mallard (*Anas platyrhynchos*) ducks. *Avian Pathol.* 38 457–467. 10.1080/03079450903349147 19937535

[B33] van Tol S. Hage A. Giraldo M. I. Bharaj P. Rajsbaum R. (2017). The TRIMendous Role of TRIMs in Virus-Host Interactions. *Vaccines (Basel)* 5:23. 10.3390/vaccines5030023 28829373 PMC5620554

[B34] Vijayakumar P. Mishra A. Ranaware P. B. Kolte A. P. Kulkarni D. D. Burt D. W. (2015). Analysis of the crow lung transcriptome in response to infection with highly pathogenic H5N1 avian influenza virus. *Gene* 559 77–85. 10.1016/j.gene.2015.01.016 25592823

[B35] Wang P. Xu J. Wang Y. Cao X. (2017). An interferon-independent lncRNA promotes viral replication by modulating cellular metabolism. *Science* 358 1051–1055. 10.1126/science.aao0409 29074580

[B36] Wang Q. Li Q. Liu R. Zheng M. Wen J. Zhao G. (2016). Host cell interactome of PA protein of H5N1 influenza a virus in chicken cells. *J Proteom.* 136 48–54. 10.1016/j.jprot.2016.01.018 26828018

[B37] Wang Q. Zhang Q. Zheng M. Wen J. Li Q. Zhao G. (2021). Viral-host interactome analysis reveals chicken stau2 interacts with non-structural protein 1 and promotes the replication of H5N1 avian influenza virus. *Front. Immunol.* 12:590679. 10.3389/fimmu.2021.590679 33968009 PMC8098808

[B38] Westermann A. J. Förstner K. U. Amman F. Barquist L. Chao Y. Schulte L. N. (2016). Dual RNA-seq unveils noncoding RNA functions in host-pathogen interactions. *Nature* 529 496–501. 10.1038/nature16547 26789254

[B39] Westermann A. J. Gorski S. A. Vogel J. (2012). Dual RNA-seq of pathogen and host. *Nat. Rev. Microbiol.* 10 618–630. 10.1038/nrmicro2852 22890146

[B40] Wise H. M. Foeglein A. Sun J. Dalton R. M. Patel S. Howard W. (2009). A complicated message: identification of a novel PB1-related protein translated from influenza a virus segment 2 mRNA. *J. Virol.* 83 8021–8031. 10.1128/JVI.00826-09 19494001 PMC2715786

[B41] Wucher V. Legeai F. Hédan B. Rizk G. Lagoutte L. Leeb T. (2017). FEELnc: a tool for long non-coding RNA annotation and its application to the dog transcriptome. *Nucleic Acids Res.* 45:e57. 10.1093/nar/gkw1306 28053114 PMC5416892

[B42] Xiao Z. McGrew J. T. Schroeder A. J. Fitzgerald-Hayes M. (1993). CSE1 and CSE2, two new genes required for accurate mitotic chromosome segregation in *Saccharomyces cerevisiae*. *Mol. Cell. Biol.* 13 4691–4702. 10.1128/mcb.13.8.4691-4702.1993 8336709 PMC360095

[B43] Yang W. Ru Y. Ren J. Bai J. Shaozu F. Liu X. (2019). G3BP1 inhibits RNA virus replication by positively regulating RIG-I-mediated cellular antiviral response. *Cell Death Dis.* 10 946–946. 10.1038/s41419-019-2178-9 31827077 PMC6906297

[B44] Zhang W. Xie R. Zhang X. D. Lee L. T. O. Zhang H. Yang M. (2020). Organism dual RNA-seq reveals the importance of BarA/UvrY in *Vibrio parahaemolyticus* virulence. *FASEB J.* 34 7561–7577. 10.1096/fj.201902630R 32281204

